# A comparative study of reconstruction modalities after knee joint-preserving tumor resection: reconstruction with a custom-made endoprosthesis versus reconstruction with a liquid nitrogen-inactivated autologous bone graft

**DOI:** 10.1186/s13018-023-04402-3

**Published:** 2023-11-29

**Authors:** Yuan Li, Hairong Xu, Huachao Shan, Ke Ma, Weifeng Liu, Xiaohui Niu

**Affiliations:** Department of Orthopedic Oncology Surgery, Beijing Jishuitan Hospital, Capital Medical University, No.31 Xin Jie Kou East Street, Xi Cheng District, Beijing, 100035 China

**Keywords:** Malignant bone tumor, Joint-preserving resection, Custom-made endoprosthesis, Liquid nitrogen-inactivated autologous bone graft

## Abstract

**Background:**

This study evaluated the feasibility, complications, graft survival rate, and clinical outcomes of joint-preserving resection using a custom-made endoprosthesis and liquid nitrogen-inactivated autologous bone graft reconstruction in patients with malignant bone tumors around the knee joint.

**Methods:**

We retrospectively analyzed 23 consecutive patients who underwent joint preservation surgery between 2008 and 2018 at our center. The study cohort included 13 patients who underwent custom-made endoprosthesis reconstruction and 10 who underwent liquid nitrogen-inactivated autologous bone graft reconstruction. The resected bone length, distance between the resection line and the joint, intraoperative blood loss, operation time, complications, and MSTS were compared between the two groups.

**Results:**

The median follow-up time was 68.5 months in the endoprosthesis group and 65.3 months in the inactivated autograft group. There were no significant differences in baseline characteristics, resected bone length, distance between the resection line and the joint, or intraoperative blood loss between the two groups. The operative time was longer in the inactivated bone graft group than in the endoprosthesis group (*p* < 0.001). The endoprosthesis group had more complications (six patients) and reoperations due to complications (five) than the inactivated autograft group (one), but there was no significant difference in the incidence of complications between the two groups (*p* = 0.158). The inactivated autograft group had one patient with type 1b complications, while the endoprosthesis group had one with type 1b complications, one with type 2b complications, and one with type 4a complications. One patient in the endoprosthesis group with type 5a complications experienced two soft tissue recurrences. The overall 5-year survival rate was 86.5% and the graft survival and final limb salvage rates were 100% in both groups. After the follow-up period, the mean MSTS scores were 91% ± 7% in the endoprosthesis group and 94% ± 6% in the inactivated autograft group, with no significant difference (*p* = 0.280).

**Conclusion:**

Joint-preserving resection is a reliable and effective tumor resection method that can achieve good postoperative function. There were no significant differences in the incidence of complications, overall survival rate, or graft survival rate between the two groups.

## Background

Malignant bone tumors preferentially develop at the metaphyses that surround the knee joint, and surgery usually involves the removal of the entire metaphysis followed by reconstruction. Currently, the most common surgical approach is reconstruction of the resected bone defect and contralateral articular surface using an endoprosthesis [1, 2]. However, for some patients, if tumor resection is performed based on preoperative imaging assessment, it is possible to obtain a safe surgical margin while preserving the articular surface, resulting in greater postoperative function. Preservation of the joints and periarticular ligaments allows for better proprioception [3]. Furthermore, the absence of a polyethylene spacer and rotatable shaft of the endoprosthesis shaft at the joint can reduce abrasive debris and mechanical complications. In pediatric patients, preservation of the epiphysis preserves the potential growth capacity of the diseased bone. The absence of a contralateral endoprosthesis also preserves the growth capacity of the epiphysis on the contralateral side of the joint [4]. For these patients, many physicians attempt to preserve the joint and use various modalities to reconstruct bone defect [4–7].

To date, the most commonly used reconstruction modalities for knee joint preservation surgeries include custom-made endoprostheses (traditional customized endoprosthesis or three-dimensional (3D) printed endoprosthesis), bone allografts, inactivated bone autografts, and allografts or inactivated autografts coupled with a free vascularized fibular graft [5–9]. Regardless of the type of reconstruction, when the thickness of the residual articular bone is small (less than 3 cm), its connection with the reconstructed part and its long-term stability are challenges for orthopedic surgeons [10]. Currently used fixation methods include screws, conventional steel plates, customized steel plates, and 3D-printed endoprostheses [4, 6, 11]. However, because only a small number of patients are suitable for this type of surgery, most published reports have small sample sizes and apply only a single method. To our knowledge, no studies have compared the results of different reconstruction modalities to date.

Our institution adopted reconstruction using both custom-made endoprosthesis and liquid nitrogen-inactivated autologous bone graft for knee joint-preserving tumor resection, and we have obtained satisfactory clinical prognosis and complication rates. However, no specific study has compared the complications, survival rate, and long-term function of these two reconstruction modalities, and there is no standardized reconstruction protocol for preserving the knee joint in specific patients. This study compared reconstruction using a customized prosthesis and a liquid nitrogen-inactivated autologous bone graft in patients who underwent knee joint-preserving tumor resection and investigated differences in (1) oncologic safety, (2) rate of complications, and (3) 5-year graft survival rate and patient limb salvage rate between the two reconstruction modalities.

## Materials and methods

### Study subjects

This study was approved by the Ethics Committee of the Beijing Jishuitan Hospital, Capital Medical University. This study retrospectively analyzed the clinical data of 23 consecutive patients with malignant bone tumors around the knees who were admitted to our hospital between November 2008 and November 2018. The inclusion criteria were as follows: (1) primary malignant bone tumors around the knee joint, (2) patients who underwent tumor resection with preservation of the knee joint, (3) residual host bone adjacent to the knee joint of ≤ 3 cm after tumor resection, and (4) reconstruction with a custom-made endoprosthesis or a liquid nitrogen-inactivated autologous bone graft. The exclusion criteria were as follows: (1) bone metastases, (2) non-first-time surgical patients, (3) patients who underwent reconstruction for non-tumor factors, and (4) patients who underwent reconstruction using a liquid nitrogen-inactivated autologous bone graft together with an autologous iliac or fibular graft. According to the above inclusion and exclusion criteria, 23 patients were included in this study cohort. They were then divided based on the reconstruction modality into a custom-made endoprosthesis group and a liquid nitrogen-inactivated autologous bone graft group.

There were 13 patients in the group with a custom-made endoprosthesis (referred to as the endoprosthesis group), including seven males and six females, with an average age of 26.3 ± 12.9 years (range: 13–49 years). There were eight patients with classic osteosarcoma, three with chondrosarcoma, one with undifferentiated high-grade pleomorphic sarcoma, and one with spindle cell sarcoma. There were 11 patients with tumors located in the distal femur and two with tumors located in the proximal tibia.

There were ten patients in the liquid nitrogen-inactivated autologous bone graft group (referred to as the inactivated autograft group), including five males and five females, with an average age of 18.74 ± 9.6 years. There were seven patients with classic osteosarcoma, one with high-grade surface osteosarcoma, one with chondrosarcoma, and one with Ewing’s sarcoma. There were five patients with tumors located at the distal femur and five with tumors located at the proximal tibia.

The difference in age between the two groups was insignificant (nonparametric Mann–Whitney U test: *Z* =  − 1.833, *p* = 0.067). There was also no significant differences in sex (*X*^2^ = 0.034, *p* = 1.000) nor the classification of diseases (*X*^2^ = 4.756, *p* = 0.446) between the two groups.

Prior to this study, we did not use a standardized method to choose between the two reconstruction modalities. Before 2015, we used only custom-made endoprostheses for reconstruction, whereas we began using liquid nitrogen-inactivated autologous bone grafts to perform reconstruction after 2015. Therefore, the patients included in this study were not randomly selected between the two reconstruction modalities. However, we analyzed the demographic characteristics of the patients and found no significant differences in patient characteristics between the two study groups.

### Preoperative preparation

All patients underwent preoperative histological testing to confirm diagnosis. Patients with osteosarcoma, undifferentiated high-grade pleomorphic sarcoma, and Ewing sarcoma received four courses of neoadjuvant chemotherapy and underwent surgery after completion of chemotherapy. The remaining patients underwent direct surgery. All patients underwent preoperative radiography, enhanced computed tomography (CT), magnetic resonance imaging (MRI), and bone scan. Both the response to neoadjuvant chemotherapy and extent of the tumor were determined based on the imaging results. If the resection was performed more than 1 cm outside the extent of the tumor and a residual bone of 10 mm could be preserved on the tibial side, or a residual bone of 20 mm could be preserved on the femoral side, knee joint-preserving tumor resection was performed.

The patients in the endoprosthesis group were treated by the attending physicians who determined the position of resection by importing the preoperative imaging data into a medical image processing software (Mimics 15.0, Mimics®; Materialize, Leuven, Belgium), followed by transmitting the Mimics data to the endoprosthetics engineers (LDK Co., Ltd., Beijing, China) for designing the prostheses. The prosthesis surface adjacent to the resection surface on the articular side must completely match the host bone resection surface. Auxiliary steel plates were designed on both sides of the prosthesis to fix the residual bone in place, and the direction and length of the screws were designed according to the size and shape of the residual bone so that there were three screws on each side. Each prosthesis was fabricated using a subtractive manufacturing method after the design was approved by the surgeon.

### Surgical procedure

Each patient underwent surgery in the supine position with an anteromedial incision at the knee joint, and the knee joint was separated and exposed outside the reaction zone following the principles of a tumor-free operation. A tracker was fixed to a safe portion of the diseased bone, and the tracker and various tools were registered at the workstation of the navigation system. Intraoperative data on the surgical area were acquired using ISO-C (Siremobil ISO-C 3D; Siemens Medical Solutions, Erlangen, Germany) and imported into the navigation system, to determine the actual resection position during surgery based on the preoperatively planned resection position. The bone was resected using a Gigli saw, and a small amount of tissue was removed from the medullary cavity of the resected end for pathological examination to determine whether a safe surgical margin had been reached.

**Fig. 1:**
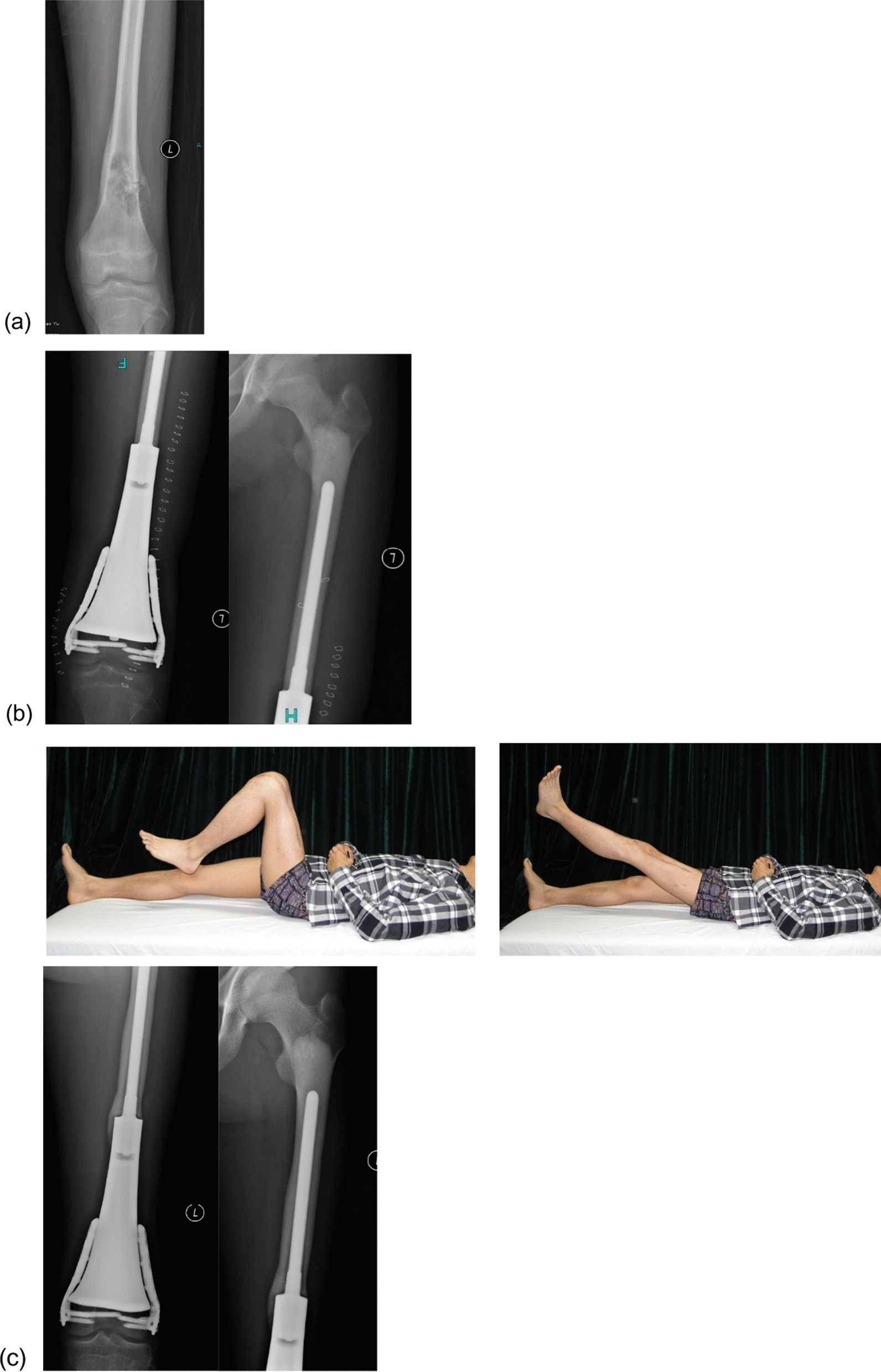
**a** A 17-year-old male with classical osteosarcoma of the distal femur. **b** Postoperative radiograph at 2 weeks. **c** Radiograph and function at 80 months postoperatively, showing the satisfactory function of the patient

In the endoprosthesis group, the defect was reconstructed using a customized endoprosthesis. The residual bone was fixed using an extracortical fixation plate after completely fitting the resection surface close to the articular side of the customized prosthesis. Intraoperative fluoroscopy was used to verify whether the direction and length of the screws matched those in the preoperative design. An intramedullary stem fixed with bone cement was placed at the other end of the prosthesis. For patients with tibial tumors, the patellar tendon was fixed to the prosthesis, and a gastrocnemius muscle flap was used to cover the anterior part of the prosthesis, whereas the wound was directly closed for the remaining patients (Fig. 1).

**Fig. 2:**
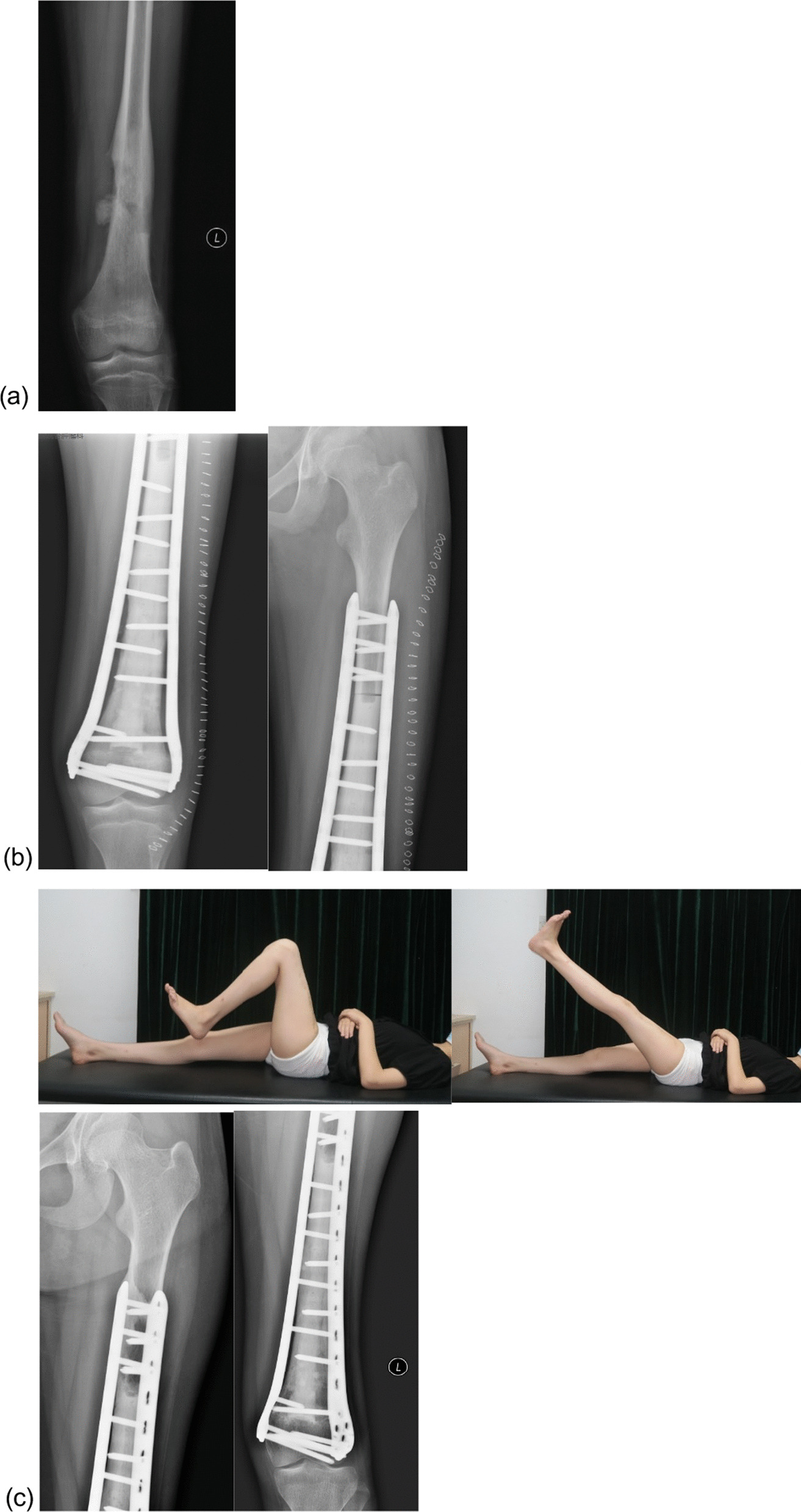
**a** A 13-year-old female with classical osteosarcoma of the distal femur. **b** Postoperative radiograph at 2 weeks. **c** Radiograph and function at 55 months postoperatively, showing satisfactory function of the patient

In the inactivated autograft group, after tumor resection, the soft tissues and muscle attachments on the surface of the bone with the tumor were removed, followed by removal of the tissues in the medullary cavity and preservation of only the cortical bone. The bones were frozen for 20 min in liquid nitrogen, rewarmed at room temperature for 15 min, and then rewarmed in saline for 15 min. The medullary cavity was then filled with bone cement. Bilateral plate fixation was performed with at least four screws bilaterally passing through the host cortical bone at the diaphysis and at least two screws on each side close to the articular side to fix the residual bone. If no suitable plate is available, the residual bone can be fixed longitudinally by using screws to avoid the weight-bearing part of the articular surface during fixation. In patients with tibial tumors, the medial head of the gastrocnemius muscle flap was used to cover the grafted bone, whereas the wound was directly closed in the remaining patients (Fig. 2).

### Postoperative rehabilitation

The surgical drain was removed when the drainage volume was less than 50 mL per day. Isometric muscle contractions were performed in the early postoperative period and joint exercises were started two weeks after surgery. Partial weight-bearing exercises were initiated six weeks after surgery. Finally, full weight-bearing exercises were started three months after surgery for patients in the prosthesis group but were started after bone healing for patients in the inactivated autograft group. Patients with osteosarcoma and Ewing sarcoma continue to receive adjuvant chemotherapy after wound healing.

### Follow-up and indicators for evaluation

Perioperative complications were documented, and outpatient follow-up visit were conducted every three months for three years after surgery and every six months thereafter. Clinical and imaging evaluations were performed at each follow-up visit to document complications and Musculoskeletal Tumor Society (MSTS) scores. The International Society of Limb Salvage (ISOLS) assessment system was used to assess graft-related complications related to the grafts [12].

In the inactivated autograft group, bone healing was assessed using the ISOLS allograft radiographic evaluation system [13]. This evaluation system was initially designed for allograft transplantation, but there have been many reports of its practical applications in imaging evaluation after autograft transplantation [14, 15]. Two senior orthopedic oncologists performed separate assessments. Any discrepancies in the results were resolved after a joint discussion.

### Statistical analysis

Statistical analyses were performed using SPSS software package (version 22.0; SPSS, Chicago, IL, USA). The categorical variables were reported using frequency and percentage, whereas the continuous variables were reported using mean ± standard deviation. Kaplan–Meier curves were used to analyze the patient and graft survival rates. The chi-square test was used to compare categorical variables, whereas a nonparametric test was used to compare continuous variables between the two groups. Statistical significance was set at *p* < 0.05.

## Results

### General results

All the patients successfully completed the surgery. For the 13 patients in the endoprosthesis group, the length of the resected bone was 91–356 mm, with an average of 188.8 ± 73.6 mm, and the distance between the resection line and the joint was 16–30 mm, with an average of 25.1 ± 4.4 mm. The duration of surgery was 180–390 min (average, 258.5 ± 54.7 min. The volume of intraoperative bleeding ranged from 150 to 1000 mL, with an average of 634.6 ± 296.8 mL. The median postoperative follow-up time was 68.5 months (20.9–177.3 months), with an average of 83.5 ± 44.7 months (Table 1).

**Table 1:** Comparison of the data between the prosthesis and inactivated autograft group

		Prosthesis group	Inactivated autograft group	p value
Number of patients		13	10	
Sex	Male	7	5	*Χ*^2^ = 0.034, *p* = 1.000
	Female	6	5	
Age		26.3 years ± 12.9 years	18.74 years ± 9.6 years	*Z* = − 1.833, *p* = 0.067
Disease classification	Classic osteosarcoma	8	7	*Χ*^2^ = 4.756, *p* = 0.446
	Chondrosarcoma	3	1	
	Undifferentiated high-grade pleomorphic sarcoma	1	0	
	Spindle cell sarcoma	1	0	
	High-grade surface osteosarcoma	0	1	
	Ewing sarcoma	0	1	
Length of resected bone		188.8 ± 73.6 mm	193.8 ± 55.6 mm	*Z* = − 0.310, *p* = 0.756
Distance between the resected bone on the articular side and the knee joint		25.1 ± 4.4 mm	22.9 ± 7.8 mm	*Z* = − 0.347, *p* = 0.729
Duration of operation		258.5 ± 54.7 min	400.0 ± 93.4 min	*Z* = − 3.578, *p* < 0.001
Volume of intraoperative bleeding		634.6 ± 296.8 mL	660.0 ± 298.9 mL	*Z* = − 0.190, *p* = 0.850

For the ten patients in the inactivated autograft group, the length of resected bone was 125–283 mm, with an average of 193.8 ± 55.6 mm, and the distance between the resection line and the joint was 10–30 mm, with an average of 22.9 ± 7.8 mm. The duration of the operation was 295–600 min, with an average of 40.0 ± 93.4 min. The volume of intraoperative bleeding was 200–1000 mL, with an average of 660.0 ± 298.9 mL. The median postoperative follow-up time was 65.3 months (13.4–86.7 months), with an average duration of 60.5 ± 21.4 months.

There was no significant difference in the length of the resected end (*Z* =  − 0.310, *p* = 0.756), distance between the resection line and the joint (*Z* =  − 0.347, *p* = 0.729), and volume of intraoperative bleeding (*Z* =  − 0.190, *p* = 0.850) between the two groups. However, the duration of operation for the inactivated autograft group was significantly higher than that of the prosthesis group (*Z* =  − 3.578, *p* < 0.001).

### Oncological results

Among the 23 patients, 46 resected ends were pathologically examined separately, and all resected ends were safe. There was no recurrence at the end of resection after surgery.

There was only one patients with local recurrence in the prosthesis group. The patient had stage III chondrosarcoma of the distal femur accompanied by pulmonary metastasis. The patient presented with two local soft tissue recurrences at 8 and 24 months postoperatively and underwent local tumor resection. Eventually, the patient died of pulmonary metastasis 30.5 months after the surgery.

Two deaths occurred in the inactivated autograft group; both patients had classic osteosarcoma of the distal femur. The patients died of lung metastases 13 and 33 months after surgery.

**Fig. 3:**
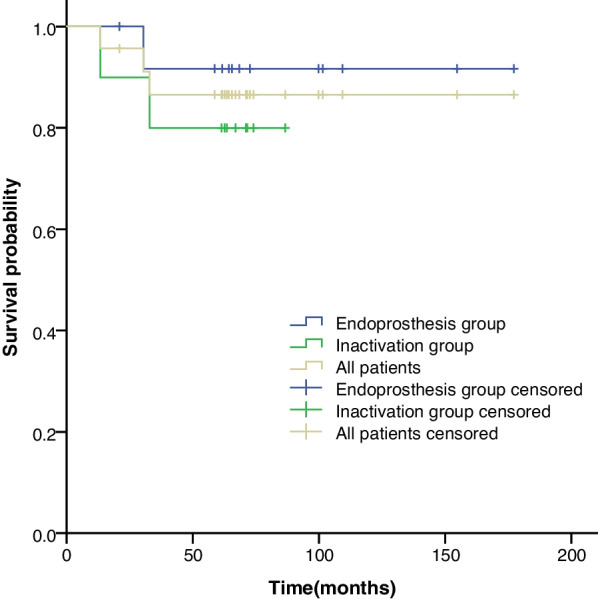
Kaplan–Meier curves showing the five-year survival rates for all the patients, the endoprosthesis group, and the inactivated autograft group. The graph shows an insignificant difference in the five-year survival rates between the endoprosthesis group and the inactivated autograft group, which were 91.7% and 80%, respectively

The 5-year survival rates were 86.5% for all patients, 91.7% in the prosthesis group, and 80% in the inactivated autograft group (Fig. 3). The difference in the survival rate between the two groups was insignificant (*X*^*2*^ = 0.639, *p* = 0.424).

### Complications

According to the ISOLS criteria for the evaluation of complications, there were six complications (26.1%) and five reoperations due to complications in the prosthesis group, whereas there was one complication and no reoperation due to complications in the inactivated autograft group. However, there was no significant difference in the incidence of complications between the two groups (*X*^*2*^ = 3.489, *p* = 0.158).

There were three patients with type 1b complications in the study cohort: two in the endoprosthesis group (one with a tumor at the distal femur and one with a tumor at the proximal tibia) and one in the inactivated autograft group (tibial tumor). All three patients developed necrosis at the skin edge due to high postoperative skin tension, which healed after debridement.

One case of aseptic loosening (type 2b) occurred in a patient in the endoprosthesis group who had an osteosarcoma at the distal femur. At the follow-up visit 52 months after surgery, imaging revealed loosening of the proximal prosthetic shaft end. Owing to the fact that the patient was asymptomatic, the patient continued using the prosthesis. No aseptic loosening of the prosthetic shaft was observed in any of the remaining patients during the follow-up.

Among the 13 patients in the endoprosthesis group, fixation of the residual bone close to the articular side of the knee joint remained stable during the follow-up period, and none of the patients had displacement of the residual bone from the prosthesis. Among the 10 patients in the inactivated autograft group, the resected end close to the knee joint side healed 6–16 months after surgery, with an average healing time of 8.0 ± 3.5 months, whereas the resected end close to the diaphysis side healed 6–26 months after surgery, with an average healing time of 13.7 ± 6.4 months. No type 2 or 3 complications related to mechanical strength were observed during follow-up.

During the follow-up period, one case of deep infection (type 4a) occurred in a patient from the prosthesis group who had osteosarcoma at the proximal tibia. A sinus tract appeared next to the anterolateral tibial incision 45 months after surgery, which healed after debridement and reappeared 53 months after surgery. The sinus tract was followed-up with intermittent changes in medication for up to 62 months.

One patient in the endoprosthesis group had two soft tissue recurrences (type 5a), and the recurrent foci were surgically resected on two separate occasions (see Oncological Results for details).

### Final graft results

No patients in either group underwent endoprosthesis or bone graft removal due to complications, and the graft survival rate was 100%. Moreover, none of the patients in either group underwent amputation due to complications, and the final limb salvage rate was 100%.

At the end of the follow-up period, the MSTS scores ranged from 80 to 100% for the prosthesis group, with an average of 91% ± 7%, whereas the MSTS scores ranged from 87 to 100% for the inactivated autograft group, with an average of 94% ± 6%. There was no significant difference between the two groups (*Z* =  − 1.081, *p* = 0.280).

## Discussion

Knee joint-preserving tumor resection for malignant bone tumors around the knee allows better function and helps reduce leg length discrepancy in minor patients in the distant future by preserving the growth capacity of the epiphysis [16, 17]. However, no standard reconstruction modality exists because of the rarity of cases that qualify for joint preservation. To provide a reference for the future selection of treatment modalities for knee joint preservation surgery, we retrospectively analyzed reconstruction using a customized prosthesis and reconstruction using a liquid nitrogen-inactivated autologous bone graft in terms of surgical difficulty, complications, and MSTS score.

Safety is the most important indicator of limb salvage surgery for bone malignancies [18]. The resection area that reaches a safe surgical margin is of utmost importance in reducing local tumor recurrence. According to Kawaguchi, the surgical margin of high-grade sarcomas must be greater than 3 cm in the absence of neoadjuvant therapy or when neoadjuvant therapy is ineffective, whereas it must be 2 cm when neoadjuvant therapy is effective [19]. All patients in this study, except for three patients with chondrosarcoma, underwent standardized neoadjuvant chemotherapy. Therefore, the resection site was designed to be > 2 cm from the tumor at the end and distal to the articular side. However, on the articular side, the minimum distance between the resection site and tumor was designed to be only 1 cm to preserve the thicker residual bone. Deng reported that in knee preservation limb salvage surgery for osteosarcoma, the difference between the resection position and the preoperatively planned position was 8.3 ± 6.0 mm when the resection was performed with bare hands, whereas the error was only 2.0 ± 1.6 mm when the resection was performed with positioning using a computerized navigation system [20]. Studies on pelvic resection and special resection surfaces have also confirmed that the accuracy of resection with computerized navigation systems is much higher than that of bare-handed resection [21, 22]. Wong also reported the use of computer-assisted positioning in joint preservation surgery for resection 1 cm from the tumor to obtain safe margins [6]. To ensure the safety of the resected bone on the articular side, all patients in this study underwent preoperative radiography, enhanced CT, enhanced MRI, and electroconvulsive therapy. The tumor margins were determined by combining the results of all examinations. During the surgery, a computer-assisted navigation positioning system was used to determine the resection position. After the intraoperative resection, the resected ends were observed to be safe with the naked eye. Tissues of the resected ends were obtained during surgery for pathological examination, and the results were all negative. There were no cases of recurrence at the end of resection during the follow-up period. There was only one case (4.3%) of soft tissue recurrence, which is comparable to the recurrence rates reported for other tumor resections around the knee (2.4–10.5%) [23–26]. Based on our experience, within a median follow-up period > 60 months, joint preservation tumor resection is a safe and acceptable approach from the perspective of oncology.

In addition to tumor safety, the precise resection position is associated with reconstruction. The two reconstruction modalities do not have the same requirements for resection position accuracy. For reconstruction with a custom-made endoprosthesis, the resection surface close to the joint affects the position and orientation of the endoprosthesis, position of the lateral steel plate, and orientation and length of the fixation screws. Therefore, the resection position must be sufficiently accurate to perfectly match the endoprosthesis. In contrast, the only requirement for the inactivation technique is that the resection position exceeds the designed safety range, making surgical resection much easier.

For joint preservation surgery, it is challenging to stabilize and fix the residual bone close to the joint to an endoprosthesis or inactivated bone and to achieve long-term stability. Reconstruction with a short-stem interpositional endoprosthesis can be used in cases of long residual bone on the articular side. Tsuda et al. reported that a custom-made endoprosthesis with a short intramedullary stem and lateral steel plate can be used for reconstruction when the resected bone is 5 cm away from the joint, and a hydroxyapatite (HA) coating is used on the surface of the endoprosthetic stem and steel plate to minimize the risk of aseptic loosening [27]. Kong reported the use of a customized prosthesis and lateral plate to fix the residual bone, and HA coating was used to enhance osseointegration at the bone-prosthesis interface [17]. Zhao et al. treated five patients with an average residual bone of 2.65 cm at the proximal or distal tibia after tumor resection with a 3D-printed porous endoprosthesis. All patients achieved early biological fixation, with an average time to clinical osseointegration of three months at the bone-prosthesis interface of 3.2 months [11]. In this study, HA coating and 3D printing technology were not used on the customized prostheses, residual bone contact surfaces, or extracortical steel plates, but there was no displacement of the residual bone from the prosthesis and no fixation failure during the follow-up period, and the five-year survival rate of the prostheses was 100%. This may be attributed to the precise design and manufacturing of the prosthesis after the surgeon has determined the resection position based on preoperative imaging, and the position and length of the internal fixation screws were determined before surgery. Moreover, a computerized navigation system was used to determine the resection position during surgery so that the endoprosthesis perfectly matched the residual bone. Six screws on both sides also provided immediate postoperative stabilization. If endoprosthesis manufacturing companies offer HA coatings for resection surfaces or 3D-printed porous structures for contact surfaces in the future, better early- and long-term stability may be achieved.

Long-term survival of liquid nitrogen-inactivated autologous bone grafts requires healing of the host bone. Commonly used inactivation methods include freezing, pasteurization, and irradiation [14, 28–30]. The liquid nitrogen inactivation method used in this study is simple, inexpensive, and does not rely on special equipment, such as radiotherapy or strict thermal control. Inactivated bone retains good biomechanical strength [31], preserves bone morphogenetic protein (BMP) [32], and retains osteoinductive capacity; however, the healing time of inactivated bone remains much longer than that of normal fractures. Wu reported that 84 patients who underwent surgery using the liquid nitrogen inactivation method achieved 79.8% healing at 18 months after surgery [14]. Araki reported that the healing time of 37 patients after surgery using the liquid nitrogen inactivation method was 3–24 months [30]. In the inactivated autograft group, the longest healing time was 16 months on the articular side and 26 months on the diaphyseal side. Therefore, it is necessary to provide an internal fixation with sufficient strength before healing. Frisoni‘s study [33] of reconstruction of backbone defect with bone allograft suggested that steel plate fixation was superior to intramedullary fixation for increasing the strength of reconstruction, which is supported by the findings of Chen et al. using inactivated bone grafts [34]. In order to provide stronger internal fixation, for all the patients in the inactivated autograft group in this study, the inactivated bone marrow cavity was filled with bone cement and then fixed with steel plates on both sides. No type 2 or 3 complications due to insufficient bone strength occurred during the follow-up.

In this study, there was no significant difference in the MSTS scores at the final follow-up visit between the two groups, which were 91% and 94% higher than the MSTS scores for conventional prostheses (71–88.6%) [9, 24, 35, 36]. Previous reports on joint preservation surgery have shown that higher MSTS scores (90–96.7%) are associated with maximum intraoperative preservation of the cruciate ligaments, medial and lateral collateral ligaments, and contralateral articular surfaces [4, 6, 37].

Based on our clinical experience, we have become familiar with the advantages and disadvantages of using custom-made endoprostheses and liquid nitrogen-inactivated autologous bone grafts for the reconstruction of preserved joints. The endoprosthesis group was not limited by the degree of osteolysis and had a shorter operation time, faster postoperative recovery, early weight bearing, and good postoperative function. However, the design and manufacturing of prostheses requires professional skills, specialized equipment, and good medical–industrial integration. Bone cement is currently used to fix prosthetic stems, which have a high long-term risk of loosening. Inactivated bone grafts require simple equipment, have a high healing rate, function well, have a low complication rate, and can be restored using an endoprosthesis after failure. However, they are not suitable for patients with severe osteolysis or pathological fractures. Moreover, there are many steps for inactivation, rewarming, and fixation of the graft during surgery, resulting in a long duration of surgery, which might increase surgical complications. Inactivated bone grafts also have slow postoperative recovery and can only bear weight after complete healing. Therefore, liquid nitrogen-inactivated autologous bone grafts are preferred for patients suitable for joint preservation surgery. Reconstruction with a custom-made endoprosthesis was performed if 50% of the cortical bone was lysed or a pathological fracture was present.

This study had some limitations. First, this was a retrospective study with a small sample size, and the two patient groups were not randomized. Second, as the liquid nitrogen inactivation method started in 2015 at our institution, all patients underwent reconstruction with a custom-made endoprosthesis before that. Third, reconstruction with a bone allograft is another possible treatment modality for joint preservation in addition to endoprosthesis and inactivated autologous bone grafts for joint preservation surgery. However, as there is no bone bank in our medical institution, matching bone allografts were difficult to obtain; therefore, reconstruction using bone allografts was not included in this comparative study. Fourth, aseptic loosening of the endoprosthesis and fracture of the inactivated bone graft increase with time, which may result in reconstruction failure [38]. The current follow-up period is still relatively short, but we will continue to follow-up and obtain 10-year survival data for patients and grafts.

For future studies, we have three main directions: (1) a multi-center collaboration to increase the allograft group for comparative analysis; (2) the use of 3D printed porous interfaces for cases using endoprosthesis to further increase their stability; and (3) longer follow-up to obtain data on graft survival and complications in long-term follow-up.

## Conclusions

This comparative study of different reconstruction modalities after knee joint-preserving tumor resection demonstrated its feasibility and safety. The use of a custom-made endoprosthesis and liquid nitrogen-inactivated autologous bone graft resulted in successful joint preservation and improved postoperative function. Although the endoprosthesis group had slightly better MSTS scores, the difference was not statistically significant. While complications during follow-up were less frequent in the inactivated autograft group, this difference was also not statistically significant.

Although this study has limitations, such as its retrospective design and small sample size, it provides valuable insights into the benefits and limitations of different reconstruction modalities. Further studies with larger sample sizes and longer follow-up periods are needed to confirm these findings and evaluate long-term patient and graft survival. This study also provides clinicians with directions for further research to optimize surgical techniques and improve patient outcomes.

## Data Availability

The datasets used and/or analyzed in the current study are available from the corresponding author upon reasonable request.

## Abbreviations

MSTS: Musculoskeletal Tumor Society
ISOLS: The International Society of Limb Salvage
